# Giant cell arteritis and scleritis: A rare association

**DOI:** 10.22088/cjim.13.3.642

**Published:** 2022

**Authors:** Abrar-Ahmad Zulfiqar

**Affiliations:** 1 Department of Internal Medicine, University Hospital of Strasbourg, 1 porte de l’hôpital, 67000 Strasbourg, France

**Keywords:** Giant cell arteritis, Scleritis, relapses, biotherapies

## Abstract

**Background:**

Giant cell arteritis (GCA) is a vasculitis of the large and medium-sized arteries in the elderly whose ischemic complications adversely affect the eye. The irreversible loss of visual acuity is most often related to acute anterior ischemic optic neuropathy. Very few cases of scleritis have been described in the literature.

**Case Presentation:**

The patient presented an obvious case of giant cell arteritis, initially revealed by an ophthalmologic involvement in the form of posterior scleritis, an ear, nose, and throat (ENT) involvement with vestibular and neurological involvement with a type of peripheral neuropathy, all evolving in the context of a weight loss of 8 kg and a marked biological inflammatory syndrome. The patient presented several relapses of giant cell arteritis in the form of several episodes of anterior and posterior, right and left, and even bilateral, isolated scleritis without any other clinical or biological abnormalities, always in conjunction with a decrease in corticosteroid therapy. In the presence of corticosteroid dependence and resistance to methotrexate, tocilizumab was initiated.

**Conclusion:**

The therapeutic management of scleritis associated with giant cell arteritis is difficult. In the absence of a codified scheme, the treatment remains empirical, based on the experience of the various teams. In this context, biotherapies (anti-IL6 type, such as tocilizumab) are increasingly used.

Giant cell arteritis (GCA), also known as temporal arteritis, is a common systemic vasculitis of medium and large-sized arteries in patients aged over 50 years with a wide spectrum of clinical manifestations. Giant cell arteritis (GCA) is a vasculitis of the large and medium-sized arteries in the elderly whose ischemic complications adversely affect the eye. They are present in 25-30% of patients with blindness in 14-18% of cases (1). Ocular manifestations are often preceded by the most common systemic symptoms of the disease: new-onset headache, scalp tender-ness, jaw claudication, and constitutional symptoms. Common ocular symptoms include visual loss, amaurosis fugax, diplopia, and eye pain. The most common ocular ischemic complication is arteritic anteriorischemic optic neuropathy with the classic sign of a pale and swollen optic disc (2). The irreversible loss of visual acuity is most often related to acute anterior ischemic optic neuropathy. Very few cases of scleritis have been described. Here, we report an original observation of a patient with recurrent scleritis in the context of giant cell arteritis.

## Case Presentation

A 65-year-old patient presented at the emergency room of a peripheral hospital for vertigo, hearing loss and tinnitus with gait disorders. 

He also complained of headaches, ear pain and a weight loss of 8 kg in the past two months, as well as lacrimation, painless palpebral edema and a slight visual blur on the right side for the past five days. He had a history of hypertension and dyslipidemia treated with sotalol and rosuvastatin.

**Figure 1:**
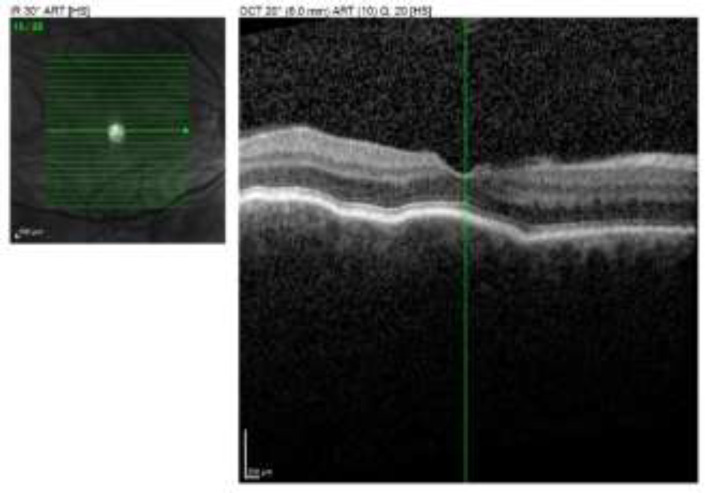
Objective choroidal folds on macular OCT (optical coherence tomography) (pictures University Hospital of Reims)

In light of the clinical assessment, he was hospitalized and was seen in inter-service consultation by the ophthalmologist who found, in particular, choroidal folds at the back of the eye as well as ocular hypotonia at 7 mmHg. The patient was then transferred to the ophthalmology department of the corresponding university hospital center. The ophthalmological examination on the left was normal. On the right, we found visual acuity at 9/10ths, palpebral edema with conjunctival hyperemia, normal photo-motor reflex, clear conjunctiva and cornea, a normal fluorescein test, no Tyndall effect (no uveitis), eye tone at 10mmHg and a narrow anterior chamber. The objective, non-dilated fundus and objective choroidal folds were also found on macular optical coherence tomography (OCT), (see figure 1). 

The cerebro-orbital CT scan performed to rule out an intra-orbital mass, came back normal, and the orbital ultrasound found a right posterior scleritis (right temporal scleral thickening of 2.7mm and a perioscleral hypoechoic border). Treatment with indocollyre three times a day for seven days and timoptol twice a day for two days was initiated.

At the same time, a general examination was also prescribed as a result of a slightly icteric complexion, the appearance of a fever and a noticeable alteration of the patient's general state. There was a major biological inflammatory syndrome with C-reactive protein (CRP) at 244 mg/L (normal rate: <5mg/L) and erythrocyte sedimentation rate at 116mm/h (normal rate: <10mm/h) as well as cytolysis and cholestasis. The patient was, after ruling out angiocholitis, referred to the internal medicine department for suspicion of giant cell arteritis with atypical signs. Corticosteroid therapy was initiated at 1 mg/kg/day with a prednisone equivalent, as well as 160mg of acetylsalicylic acid daily, (along with supplementation of vitamin D, calcium and potassium). A cerebral magnetic resonance imaging (MRI), positron emission tomography-scan (PET-scan) and thoraco-abdomino-pelvic scan came back normal. The clinical examination showed, in particular, a bilateral vestibular head impulse test syndrome and neuropathy of the lower limbs with proprioceptive impairment. The vestibular syndrome was attributed to cochleovestibular microvascularity. The patient was subsequently referred to physical medicine and rehabilitation, where he recovered a stable bipedal balance. However, a closed-eye unipodal balance disorder persisted in tandem. The left temporal artery biopsy on the left side came back positive, confirming the diagnosis of Giant cell arteritis. One month later, the scleritis had completely and spontaneously regressed, and the orbital ultrasound was normal as well as the macular optical coherence tomography (OCT). Six months later, he relapsed ophthalmologically, following the progressive decrease of corticosteroids to 8 mg/day and presented with scleritis of the left eye requiring a corticosteroid increase to 40 mg/day.

One year after diagnosis, he was hospitalized for a new relapse of anterior scleritis of the left eye due to the ineffectiveness of the local treatment prescribed by ophthalmology unit. The biological examination found a moderate biological inflammatory syndrome (neutrophilic leukocytosis at 12 Giga/L (normal rate: 4Gigal/l-10Giga/l) and a CRP at 11.6 mg/L (normal rate: <5 mg/l)). A cerebral MRI scan was performed and captures contrast images of the sclera, the left lacrimal gland and the retro-orbital fat evoking anterior scleritis of the left eye. He received 120 mg methylprednisolone for three days and was supplemented with methotrexate 10 mg/weekly. 

The corticosteroids had been administered per os at 20 mg/day with a progressive decrease to 5 mg/day, all the more so as he presented an imbalance of his hypertension and corticosteroid-induced diabetes. Three months later, he had another ophthalmologic relapse with bilateral scleritis as soon as the corticotherapy was lowered to 5 mg/day, which improved with the increase of corticotherapy to 7 mg/day in the form of anti-inflammatory eye drops. After three months, scleritis of the right eye recured. Methotrexate was increased to 20 mg/week and corticosteroids to 10 mg/day. Six months later, Methotrexate was stopped in the context of managing the prostatic adenocarcinoma. 

The disease relapsed the following month (with headache, fever, asthenia, weight loss and major biological inflammatory syndrome with CRP at 146 mg/L (normal rate: <5mg/L)) and improved dramatically under three boluses of methylprednisolone 250 mg/day for 3 days relayed by corticosteroids 20mg/day and resumption of methotrexate 10 mg/week, which was increased in the following months to 15 mg, and then to 20 mg. The second temporal artery biopsy on the right side also came back negative. In the presence of corticosteroid dependence and resistance to methotrexate, tocilizumab was initiated at 8mg/kg every 4 weeks.

## Discussion

This patient presented an obvious case of giant cell arteritis, initially revealed by an ophthalmologic involvement in the form of scleritis, an ear, nose, and throat (ENT) involvement with vestibular and neurological involvement with a type of peripheral neuropathy, all evolving in the context of a weight loss of 8 kg and a marked biological inflammatory syndrome. It should be noted that the patient had also the hepatic biological abnormalities commonly described in the context of giant cell arteritis (3). Subsequently, our patient presented several relapses of giant cell arteritis in the form of several episodes of anterior and posterior, right and left, and even bilateral, isolated scleritis without any other clinical or biological abnormalities, always in conjunction with a decrease in corticosteroid therapy. To our knowledge, very few cases have been reported in the literature (4, 5, and 6).

Scleritis is an inflammatory disease of the sclera that can be anterior, posterior or mixed (7, 8). It has rarely, if ever, been reported in the context of giant cell arteritis. The diagnosis of anterior scleritis is primarily clinical. On the other hand, certain additional examinations are often useful to confirm the diagnosis of posterior scleritis. This is particularly the case with B-mode orbital ultrasonography, which reveals a hyperechoic thickening of the posterior sclera, and with macular OCT (optical coherence tomography), which can objectify choroidal folds and sometimes a retinal serous retinal detachment, as shown in figure 1 of our observation.

In the etiological assessment of scleritis, a systemic disease is found in 60% of cases, this figure being higher for posterior scleritis (7, 8). Thus, a retrospective file-based study between 2009 and 2017 of 34 patients with scleritis or episcleritis found a systemic etiology in a total of 22 (64.7%). It should be noted that only one of the patients in this study had a scleritis whose etiology was giant cell arteritis. In a larger cohort of 500 patients with scleritis in a retrospective study conducted in Spain (8), 179 patients had a systemic pathology (35.8% of cases), of which the most represented was rheumatoid polyarthritis. In this series, only one patient had giant cell arteritis. Scleritis is therefore an uncommon manifestation during giant cell arteritis, but should alert the clinician, especially in the presence of fever, weight loss, headache and inflammatory syndrome.

As illustrated by our observations, the therapeutic management of scleritis associated with giant cell arteritis is difficult, relying on local treatments (corticosteroid-based eye drops and non-steroidal anti-inflammatory drugs), which fluctuates according to the evolution and relapses between oral corticosteroid therapy, corticosteroid boluses and immunosuppressants (especially methotrexate). In the absence of a codified scheme, the treatment remains empirical, based on the experience of the various teams. In this context, biotherapies (anti-IL6 type, such as tocilizumab) are increasingly used.
